# Impact of genetic and pharmacological modulation of CB2 receptors on morphine-induced analgesia, tolerance, and reward in C57BL/6J mice

**DOI:** 10.1016/j.ibneur.2024.10.006

**Published:** 2024-10-24

**Authors:** Machich Omar, Safae Ouzzaouit, Meriem Moussafir, Souad Skalli, Khalid Taghzouti, Oualid Abboussi

**Affiliations:** a Laboratory of Physiology and Physiopathology, Department of Biology, Faculty of Science, Mohammed V University in Rabat, Morocco; b Microbiology and Molecular Biology Team, Center of Plant and Microbial Biotechnology, Biodiversity and Environment, Faculty of Science Mohammed V University in Rabat, Morocco

**Keywords:** Morphine, CB2 cannabinoid receptors, Analgesia, Tolerance, Reward

## Abstract

The use of morphine for pain management often leads to the development of tolerance and addiction, posing significant challenges in clinical practice. This study aims to achieve sustained morphine-induced analgesia while mitigating tolerance and reward development by targeting CB2 receptors. In this investigation, male and female C57Bl/6 mice, both wild-type and CB2 knockout, received various doses of morphine or vehicle daily over 10 days. Wild-type mice were also administered a CB2 antagonist (SR144528) prior to morphine injection to assess CB2 receptor involvement. Analgesic effects were evaluated using tail flick tests, while conditioned place preference tests measured reward effects. Our results demonstrate that wild-type mice developed tolerance to morphine within 6 days, whereas CB2 knockout mice showed sustained analgesia throughout the study period. Combining SR144528 with morphine prevented tolerance development, maintaining efficacy even at higher doses. Additionally, CB2 knockout mice exhibited increased sensitivity to morphine reward. Overall, genetic and pharmacological manipulation of CB2 receptors reduced tolerance but exacerbated drug-seeking behavior.

## 1 Introduction

Opioid addiction has emerged as a serious global concern, with the World Health Organization (WHO) attributing half a million deaths to drug use. Of these fatalities, over 70 % are associated with opioids and more than 30 % result from overdose (WHO, 2023).

The physiological response to pain begins with activating nociceptors throughout the body (Dubin and Patapoutian, 2010). These pain signals travel via type C nociceptive nerves to the dorsal horn of the spinal cord, and subsequently, through the spinothalamic tract to the brain for interpretation (Todd, 2010). Following this, the production of analgesia involves the activation of the descending pathway, which commences with the disinhibition of the rostral ventromedial medulla (RVM) and the locus coeruleus (LC). This activation triggers the release of serotonin and noradrenaline, leading to the inhibition of GABAergic neurons and the disinhibition of opioidergic interneurons. As a result, nociceptive fibres at the dorsal horn are hyperpolarized, contributing to pain relief (Kwon et al., 2014, Bannister, 2019). Morphine, a potent analgesic, exerts its effects primarily through the activation of the Mu-opioid receptor (MOR) (Lipp, 1991). This receptor is expressed on neurons, microglia, and astrocytes in key brain regions including the periaqueductal grey (PAG), RVM, ventricular tegmental area (VTA), and nucleus accumbens (NAcc). Upon acute administration, morphine activates the descending pathway, facilitating pain relief (Martínez-Navarro et al., 2019).

While morphine remains widely utilized for pain management, its clinical efficacy is constrained by the development of tolerance and addiction (Badshah et al., 2023). Despite numerous investigations into the mechanisms underlying morphine tolerance, a comprehensive understanding remains elusive. Current research suggests several potential factors contributing to this phenomenon. These include the desensitization and internalization of the MOR, interplay with signaling pathways such as the mitogen-activated protein kinase (MAPK) pathway, the involvement of microglia, and an upsurge in inflammatory cytokines (Martini and Whistler, 2007, Bull et al., 2017). Nonetheless, further elucidation of these mechanisms is essential for enhancing the efficacy and safety of morphine-based pain management strategies. Recent literature data have shown that Mu-receptors are coexpressed with the CB1 and/or CB2 receptors in several structures, including the VTA, the epidermis, and the NAcc (Wilson-Poe et al., 2012, Maguire and France, 2014, Ozdemir, 2020). The endocannabinoid system, comprising cannabinoid receptors (CB receptors), plays a crucial role in modulating various physiological processes. CB receptors are G-protein coupled receptors activated by lipidic ligands such as Anandamide (AEA), 2-Arachidonoylglycerol (2-AG), and compounds found in *Cannabis sativa* (Cannabaceae), including cannabidiol (CBD) and Tetrahydrocannabinol (THC), along with synthetic ligands like SR144528 and AM19. Among these receptors, CB2 receptors exhibit widespread distribution throughout the body, with a significant presence in organs such as the testis, ovaries, lungs, and gastrointestinal tract, as well as in the central nervous system, particularly in astrocytes (Finn et al., 2021, Lu and Mackie, 2021).

The most recognized pharmacological effect associated with CB2 receptor activation is its anti-inflammatory properties. Additionally, CB2 receptor activation has been linked to the enhanced analgesic effects of morphine and the induction of epithelial endorphin production, contributing to analgesia (Bryk and Starowicz, 2021). Moreover, CB2 receptors play a role in modulating the expression of MOR in the brainstem of mice, suggesting a complex interplay between these receptors. The interaction between MOR and CB2 receptors involves bidirectional cross-talk, where modulation of one receptor can influence the activity of the other. For instance, the CB2 receptor agonist Noladin inhibits MOR activation, while morphine administration increases CB2 receptor density in the dorsal horn. This intricate interplay underscores the potential for targeted manipulation of the endocannabinoid system to modulate pain perception and inflammatory responses (Páldyová et al., 2008). Several Studies have suggested that inhibiting the CB2 receptors may result in decreased tolerance and reward in mice (Zhang et al., 2018, Li et al., 2019, Cui et al., 2023). Our primary goal is to investigate the intricate interplay between these two systems. To achieve this, a CB2 knockout animal model was used. On a fundamental pharmacological level, we intend to administer a CB2 receptor antagonist to elucidate their impact on nociception, tolerance, and reward responses to morphine. Through this comprehensive approach, we aim to gain a deeper understanding of the complex mechanisms underlying pain perception, tolerance development, and addictive behaviors associated with morphine use.

## 2 Methods

### 2.1 Animals

Two hundred eighty-eight male and female wild type (WT) and CB2-/- mice, bred on the C57BL/6 J background were housed in the laboratory of Physiology at the Faculty of Sciences in Rabat, Mohammed V University, following the local ethics committee regulations. The CB2-/- mice were generated by The Jackson Laboratory, Canada (RRID: IMSR_JAX:005786). They were originally purchased by the R. Diaz laboratory at the Centro de Investigación Biomédica en Red de Salud Mental (CIBERSAM), Madrid, Spain, who kindly provided them for use in this study. The animals were kept under standard laboratory conditions, with a temperature maintained at 21±1°C, a 12-hour light/dark cycle, and ad libitum access to standard chow and tap water. Before the commencement of experiments, a week-long acclimatization period was provided during which all mice were routinely handled, weighed, and familiarized with the injection procedure. Genotyping of the mice was conducted by the Moroccan National Center of Technical and Scientific Research (CNRST). All experimental procedures adhered strictly to the guidelines for the ethical treatment of animals in behavioral research (Committee, 2024).

### 2.2 Experimental protocols and drug administration

Before initiating each experiment, mice aged 7–9 weeks, encompassing both sexes males and females, underwent a habituation phase. All experimental procedures were exclusively conducted during the light phase. The animals received either vehicle or morphine sulfate (Sigma-Aldrich, UK), diluted in 0.9 % NaCl, via subcutaneous (s.c.) injection, or SR144528, a CB2 receptor inverse agonist (ab146185, Abcam, US), prepared in 1 % DMSO, administered intraperitoneally (i.p.). To ensure randomization, mice were assigned to either vehicle- or drug-treated groups while maintaining a balance between males and females.

In the tail-flick experiment, strict blinding protocols were followed to eliminate bias. Data from conditioned place preference and locomotor activity were collected and analyzed using AnyMaze software (Stoelting, US), ensuring objective and automated assessment. Sample sizes were determined based on preliminary investigations and power analysis to achieve robust statistical significance**.**

Four distinct experiments were conducted to evaluate the antinociceptive effects of morphine using various parameters observed through the tail-flick test:

− Experiment 1: To determine the effective antinociceptive dose of morphine WT (n=8) and CB2-/- (n=8) received morphine at varying doses, at least five doses ranging from 0 to 20 mg/kg. Different animals were used for each dose group, with no within-subject design applied. Thus, a total of 80 animals were utilized.
− Experiment 2: To explore morphine tolerance development, WT and CB2-/- mice (n=8 per group received daily injections of 10 mg/kg morphine for 10 consecutive days. Each group consisted of distinct animals, separate from those used in Experiment 1, totalling 16.
− Experiment 3: To investigate the antinociceptive effect of morphine in the presence of the CB2 receptor antagonist SR144528 in WT mice. Morphine was administered at doses ranging from 0 to 20 mg/kg, 30 minutes following i.p. injection of SR144528 at 3 mg/kg or vehicle (n=8 per group). Different groups received varying doses of morphine following SR144528 or vehicle administration, with 80 animals used in total, and no within-subject design.
− Experiment 4: To assess the pharmacological modulation of the CB2 receptor in tolerance to morphine, WT mice received SR144528 (n=8) or vehicle (n=8) and they were treated daily for 10 days with 10 mg/kg of morphine, totalling 16 animals.

To evaluate the impact of CB2 receptors on the rewarding effect of morphine, the Conditioned Place Preference (CPP) test was employed (Prus et al., 2011). This test assessed the effects of morphine on locomotion, the development of place preference, and reinforcement across different genotypes. Both WT and CB2-/- mice were injected with saline or morphine (3 or 10 mg/kg) (n=8 per group totalling 48 animals). Moreover, WT mice receiving SR144528 (3 mg/kg) were injected with morphine at both 3 and 10 mg/kg or vehicle (n=8 per group). Each group was distinct, and no within-subject design was employed, with a total of 48 animals used.

### 2.3 Tail-flick test

The tail-flick test was utilized to assess the role of CB2 receptors in thermal nociception, conducted in two phases. In the first phase, wild-type (WT) and CB2 knockout (CB2-/-) mice were used to evaluate the impact of CB2 receptor genetic deletion on nociceptive responses, with both genotypes receiving varying doses of morphine. In the second phase, WT mice were administered either vehicle or SR144528 (3 mg/kg, i.p.), a selective CB2 receptor antagonist (Abcam, ab146185, US), 30 minutes prior to morphine administration. Morphine sulfate (Sigma-Aldrich, UK) was administered at doses ranging from 0 to 20 mg/kg, and a log(dose) scale was applied to assess dose-response effects and calculate the ED50. To evaluate morphine tolerance, WT mice received daily injections of morphine (10 mg/kg) for 10 consecutive days. Tail withdrawal latency was measured 30 minutes post-drug administration using a hot-water bath (Thermo Fisher) maintained at a constant temperature within ± 0.1°C to ensure thermal consistency, with a maximum exposure time of 15 seconds to prevent tissue damage (Mulder & Pritchett, 2004). Baseline tail withdrawal latency was recorded for each mouse to account for individual variability. Analgesia was expressed as the maximal potential effect (MPE) percentage, calculated using the formula: MPE (%) = [(post-drug latency - baseline latency) / (cutoff latency - baseline latency)] × 100. Latency measurements were then compared across treatment groups to evaluate both antinociceptive effects and morphine tolerance development.

### 2.4 Conditioned place preference test (CPP)

In this study, we employed the Conditioned Place Preference test (CPP) to investigate the effects of morphine on locomotion, the development of place preference, and reinforcement in the WT and CB2-/-, and SR14-treated mice, which received subcutaneous injections of morphine at either 3 or 10 mg/kg, or vehicle.

The CPP test was conducted in a two-compartment apparatus, each measuring 18 ×18 x 24 cm and separated by a sliding partition. One compartment featured a white plastic mesh grid on the floor and walls with a checked white pattern, while the other compartment had a smooth black floor and plain black walls. This test was tracked by AnyMaze software.

The experiment followed three distinct phases:

1. Habituation Phase (Day 1): Mice were randomly placed in one of the two compartments and allowed 20 minutes of free access to both compartments. This phase aimed to determine each mouse's preferred compartment based on the record of the time spent in each compartment.
2. Conditioning Phase (Days 2–4): Across six sessions distributed over three days, animals underwent conditioning twice daily (morning and afternoon). Following each injection of morphine or vehicle, there was a deliberate delay of 30 minutes before the mice were placed in the CPP apparatus. Depending on the experimental group, animals received either morphine (at doses of 3 or 10 mg/kg) or vehicle solution and were then confined to their less preferred compartment for 30 minutes, or they were confined to their preferred compartment solely with the vehicle. This procedure aimed to establish an association between the effects of morphine and a specific compartment.
3. Post-Conditioning (Day 5): 24 hours following the last conditioning session, the animals were placed in the place preference apparatus and were allowed free access to both compartments for 15 min, which was regarded as the post-test. During this phase, time spent in each compartment and locomotor activity were assessed using the Any-Maze video-tracking system.

To simplify the presentation of the data, we calculated the preference score as the difference between the time spent in the morphine-associated compartment during the post-conditioning test and the time spent in the initial or least preferred compartment during the pre-test. This score provided a quantitative measure of the development of place preference in response to morphine exposure.

### 2.5 Data analysis

To evaluate the antinociceptive effects in our experiments, we used the percentage of maximum antinociceptive effects (% MPE) as the primary measurement. % MPE was computed from tail-withdrawal latencies (tail-flick test) using the following formula:

% MPE = [(test latency - baseline) / (cutoff - baseline)] × 100

In this equation, "test latency" represents the recorded latency during the test, "baseline" denotes the baseline latency, and "cutoff" is the predetermined cutoff latency. The result is then multiplied by 100 to express the percentage.

### 2.6 Statistical analysis

Statistical analyses were conducted using GraphPad Prism 8 software. Normality was assessed using the Shapiro-Wilk test. To investigate the effects of different morphine doses and the development of tolerance to morphine, as well as its effects on motor function and place preference in WT and CB2-/- mice, either a repeated-measures ANOVA with time and genotype as factors or a two-way ANOVA with morphine dosage and genotype as factors were applied. The correlation between locomotor activity and place preference was analyzed using a simple regression test. In instances of a significant interaction between these parameters, a Bonferroni post hoc test was conducted to adjust for multiple comparisons. The significance level was set at p < 0.05, and data were expressed as means ± standard error of the mean (SEM).

## 3 Results

### 3.1 Morphine efficiency and tolerance development in wild-type and CB2 receptors knockout mice

**Fig. 1:**
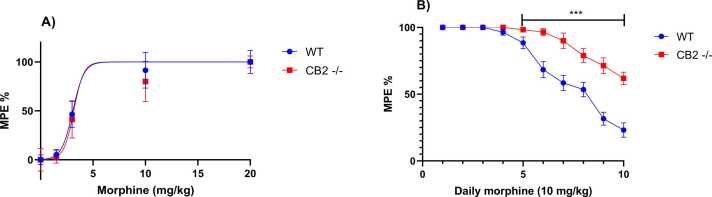
Determination of effective analgesic dose of morphine (A), and development of morphine tolerance over 10 days of daily administration of morphine (10 mg/kg) (B) in wild-type (WT) and CB2 receptor knockout mice (CB2-/-) evaluated using the tail flick test. Data were expressed as Maximum Potential Analgesic Effect (MPE)% and presented as mean ± SEM (n = 8 per group, ***p<0.001 in comparison to WT, Bonferroni post-hoc test).

To determine the effective morphine dose, we measured the antinociceptive responses of WT and CB2-/- mice across a range of morphine doses (0–20 mg/kg) using the tail-flick test. Data analysis of morphine's effect on tail-flick latency in WT and CB2-/- mice was conducted using an ANOVA test, considering genotype and morphine dose as factors. The analysis revealed a significant effect of morphine (F_(2, 31)_ = 182.4, p < 0.0001), but no significant effect of genotype (F_(1, 14)_ = 2.378, p = 0.1454) or interaction between genotype and dose (F_(4, 56)_ = 0.4937, p = 0.740) on the animals' response to morphine at different doses in the tail withdrawal test. Additionally, the IC50 was determined to be 3 mg/kg in both genotypes (see Fig. 1A). Consequently, a dosage of 10 mg/kg of morphine was selected to evaluate the development of tolerance over 10 days in both WT and CB2-/- mice.

Data analysis utilizing repeated-measures ANOVA showed a significant effect of genotype (F_(1, 14)_ = 40.25, p < 0.0001) and time (F _(9, 126)_ = 73.67, p < 0.0001) on the development of tolerance to morphine, with a notable interaction between these two factors (F_(9, 126)_ = 11.02, p < 0.0001). Bonferroni's post-hoc analysis further revealed that tolerance onset occurred more rapidly in WT mice compared to CB2-/- counterparts, becoming evident on day 5 of treatment (p < 0.05). These findings suggest a potential involvement of CB2 receptors in the development of morphine-induced tolerance (see Fig. 1B).

### 3.2 Effect of CB2 receptor inhibition on the maintenance of the analgesic effect of morphine

**Fig. 2:**
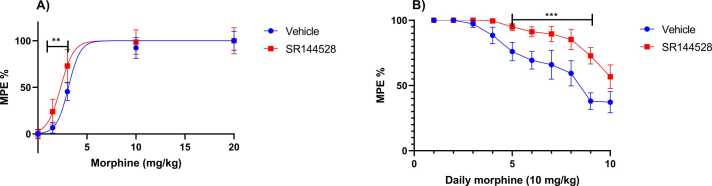
Determination of effective analgesic dose of morphine (A), and development of morphine tolerance over 10 days of daily administration of morphine (10 mg/kg) (B) in wild-type (WT) mice pre-treated with SR144528 (3 mg/kg) or vehicle 30 minutes before morphine administration, evaluated using the tail flick test. Data were expressed as Maximum Potential Analgesic Effect (MPE)% and presented as mean ± SEM (n = 8 per group, **p<0.01, ***p<0.0001 in comparison to vehicle, Bonferroni post-hoc test).

To investigate the impact of CB2 receptor inhibition on morphine-induced analgesia, WT mice were administered SR144528 at a dosage of 3 mg/kg, 30 minutes before receiving escalating doses of morphine. Data analysis using ANOVA with morphine doses and SR144528 pre-treatment revealed a significant analgesic effect of morphine (F _(4, 70)_ = 269, p < 0.0001), a significant effect of pre-treatment with SR144528 (F _(1, 70)_ = 15.42, p = 0.0002), and a significant interaction between these two parameters (F _(4, 70)_ = 4.909, p = 0.0015). The Bonferroni post hoc test showed that SR144528 pre-treatment significantly increased the sensitivity to the analgesic effect of morphine at doses of 1.5 and 3 mg/kg; however, at doses higher than 3 mg/kg, there were no significant differences (Fig. 2A).

When analyzing the impact of inhibiting CB2 receptors with SR144528 on morphine (10 mg/kg) induced tolerance, notable findings emerged. The results demonstrated a significant effect of SR144528 pre-treatment (F _(1, 14)_ = 10.64, p = 0.0057), as well as a significant effect of repeated administration of morphine (F _(3, 41)_ = 26.53, p < 0.0001), with a noteworthy interaction between these factors (F _(9, 126)_ = 2.713, p = 0.0064). Bonferroni post hoc analysis revealed significant maintenance of the analgesic effect of morphine between days 5 and 9 in animals pre-treated with SR144528, while it decreased in animals pre-treated with vehicle (Fig. 2B).

### 3.3 Effect of genetic modulation of CB2 receptors on morphine induced psychomotor stimulation and reward

**Fig. 3:**
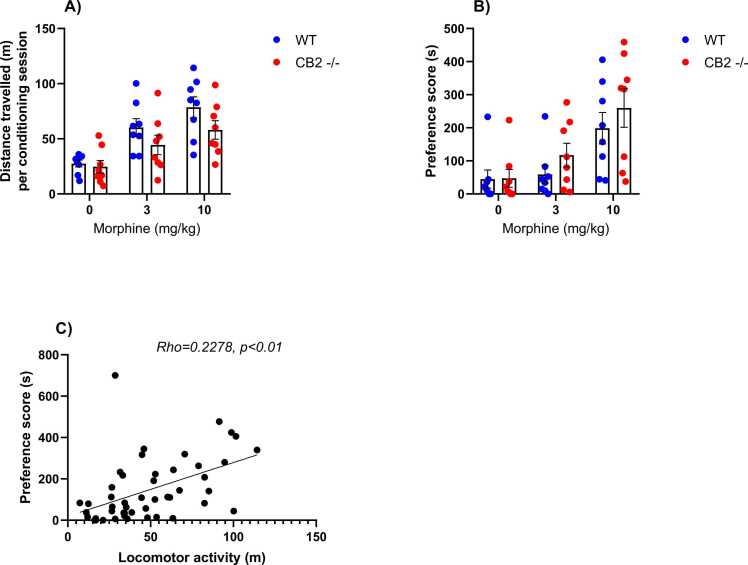
Implication of CB2 receptors in morphine-induced locomotor activity and reward. (A) Morphine-induced psychomotor stimulation at doses of 3 and 10 mg/kg, and (B) reward at a dose of 10 mg/kg during conditioning and post-conditioning test in WT and CB2-/- mice in the CPP test. (C) Positive correlation between locomotor activity and place preference (simple regression analysis). Data are presented as mean ± SEM (n=8 per group).

In addition to evaluating analgesia, we investigated morphine's psychomotor stimulatory and rewarding effects in mice using the CPP test. Data analysis unveiled a significant main effect of morphine dosage (F _(2, 42)_ = 15.81, p < 0.0001) and genotype (F _(1, 42)_ = 4.427, p = 0.0414) on locomotor activity throughout the three days of conditioning sessions in the CPP test. Notably, there was no significant interaction between these two factors (F _(2, 42)_ = 0.7242, p = 0.4907). CB2-/- mice displayed lower locomotor activity compared to WT animals in response to morphine administered at 3 and 10 mg/kg (see Fig. 3A).

**Fig. 4:**
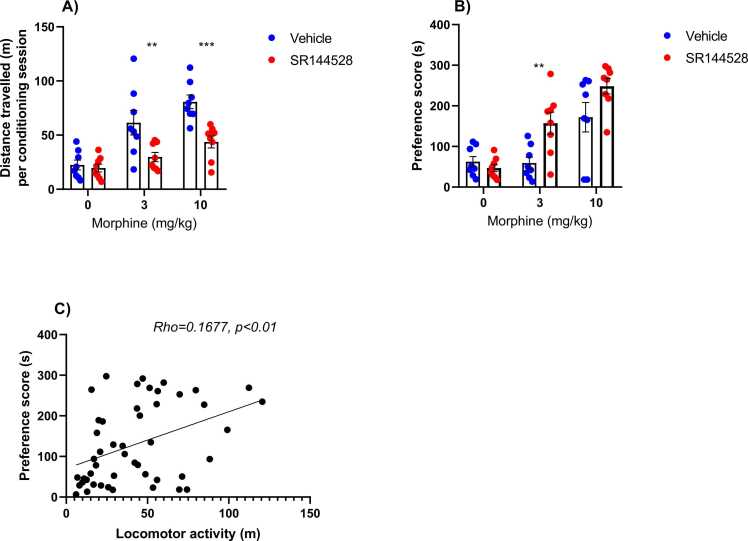
Modulatory effect of SR144528 CB2 receptors reverse agonist (3 mg/kg) on morphine-induced locomotor activity and reward. (A) SR144528 inhibits morphine-evoked psychomotor stimulatory effect at a dose of 3 and 10 mg/kg (A) and increases its rewarding effect starting from a dose of 3 mg/kg (B) during the conditioning sessions and post-conditioning test during the CPP test, respectively, in comparison to wild type mice who received vehicle. (C) Positive correlation between locomotor activity and place preference (simple regression analysis). Data presented as mean ± SEM (n=8 per group, **p<0.01, ***p<0.0001 in comparison to vehicle, Bonferroni post-hoc test).

The psychomotor stimulatory effect induced by morphine in both WT and CB2-/- mice correlated with increased place preference in both genotypes during the CPP post-conditioning test. Two-way ANOVA analysis demonstrated a significant effect of morphine dosage (F _(2, 42)_ = 12.09, p < 0.0001) on the time spent by animals in the morphine-paired compartment. Although CB2-/- mice appeared to develop a stronger preference for morphine following injection with 3 mg/kg compared to WT mice, statistical analysis revealed no significant effect of genetic modulation of CB2 receptors on the rewarding effect of morphine administered at 3 and 10 mg/kg (F _(1, 42)_ = 1.630, p = 0.2087), nor was there a significant interaction between these two factors (F _(2, 42)_ = 0.3561, p = 0.7025) (see Fig. 3B). The regression analysis revealed a significant positive correlation between locomotor activity and place preference (slope = 2.597, F = 13.57, p = 0.0006) (see Fig. 4C).

### 3.4 Effect of Pharmacological modulation of CB2 receptors on morphine induced psychomotor stimulation and reward

Pharmacological modulation of CB2 receptors using SR144528, administered at a dose of 3 mg/kg before morphine treatment, yielded significant effects on morphine-induced psychomotor activity stimulation (F _(1, 42)_ = 20.39, p < 0.0001). Morphine alone also exhibited a significant effect, increasing locomotor activity (F _(2, 42)_ = 20.63, p < 0.0001). Furthermore, statistical analysis revealed a significant interaction between pre-treatment with SR144528 and morphine (F _(2, 42)_ = 4.072, p = 0.0242). Bonferroni's multiple comparisons tests showed that SR144528 significantly decreased the distance travelled by animals following treatment with morphine at doses of 3 and 10 mg/kg during the conditioning sessions of the CPP. This underscores the role of CB2 receptor modulation in attenuating morphine-induced psychomotor stimulation (see Fig. 4A).

Moreover, during the CPP post-conditioning test, the obtained results showed a significant effect of SR144528 (3 mg/kg) pre-treatment (F _(1, 42)_ = 8.883, p = 0.0048), of morphine dosage (F _(2, 42)_ = 26.48, p < 0.0001) on place preference in the wild-type mice, and a significant interaction between these two factors (F _(2, 42)_ = 3.861, p = 0.0289), indicating that inhibition of CB2 receptors prior to morphine administration influences its rewarding effect. Interestingly, Bonferroni post hoc comparisons revealed that SR144528 increases the rewarding effect of morphine even when administered at a dose of 3 mg/kg compared to animals who received only the vehicle (see Fig. 4B). A simple regression analysis was performed to examine the relationship between locomotor activity and place preference. The regression analysis revealed a significant positive correlation between locomotor activity and place preference (slope = 1.397, F = 9.268, p = 0.0039, see Fig. 4C).

## 4 Discussion

Our results show that CB2 receptor genetic and pharmacologic modulation impact morphine efficiency, tolerance onset, and overall reward. First, we started by determining the doses needed for the study our results show that at 10 mg/kg, morphine procures a complete analgesic effect regardless of CB2 receptor status.

While morphine efficiency, decreased in WT mice on the 6th day of treatment, morphine maintained its analgesic effect in CB2-/- mice until 10 days (end of the experiment) and similarly, the use of SR144528, a CB2 receptor antagonist, produced the same effect, indicating that the CB2 receptor may play a role in morphine-induced tolerance. The studies on these subjects are extremely controversial, in the study by (Páldy et al., 2008), CB2 receptor knockout in mice drastically decreased the efficacy of DAMGO, a highly selective MOR agonist in the brainstem, this effect is also produced by SR144528 and LY2828360 (Hua et al., 2020, Tarawneh et al., 2022). To our knowledge, only one study performed by Altun et al. (2015) demonstrated that CB2 receptor inhibition decreases analgesia and decreases tolerance to morphine. Furthermore, a study by Zhang et al., using a murine model that mirrors cancer pain by injecting cancer cells (breast cancer) into mice showed that the use of CB2 receptor agonists increases morphine analgesia and decreases its tolerance (Zhang et al., 2016).

The differences in the strains of mice used in these studies might be a significant factor contributing to the varying results observed. Strains of mice can exhibit different baseline pain thresholds, genetic predispositions, and drug responses due to inherent genetic differences. For instance, certain strains may have variations in the expression or function of pain-related receptors, including CB2 receptors, which could impact how morphine and CB2 receptor modulators affect pain and tolerance. These genetic differences could lead to divergent responses to morphine and its interaction with CB2 receptor modulation. Additionally, the specific genetic background of each strain can influence the development and progression of tolerance to morphine, which might explain the discrepancies in findings between studies (Lin et al., 2018, Carey et al., 2023).

The dose administered in each study and its mode of administration may also impact the results. Different strains might metabolize or respond to morphine differently, which could affect the observed efficacy and tolerance development. For instance, strains with altered opioid receptor expression or activity might show varying degrees of analgesia and tolerance in response to morphine. Moreover, the use of different pain models, such as paclitaxel injection versus tumor cell injection, could induce varying levels of pain and inflammatory responses, further complicating the comparison of results across studies (Smith, 2019).

These pains, whether originating from cancerous growths or inflammatory conditions, trigger immune system dysfunction. This leads to an overproduction of pro-inflammatory cytokines, chemokines, and mediators, which in turn sensitizes nociceptors through increased expression or phosphorylation, enhancing their effectiveness. Consequently, there's a shift in cellular signaling dynamics (Pinho-Ribeiro et al., 2017). The CB2 receptor, known for its ability to modulate various signaling processes, likely adapts its function through alterations in expression levels or ligand interactions.

After delineating the effects of CB2 receptor modulation on morphine tolerance and analgesia, our investigation delved into its impact on reward mechanisms. Our results indicated that morphine induces psychomotor stimulation at doses of 3 and 10 mg/kg and elicits reward at a dose of 10 mg/kg during the conditioning sessions and post-conditioning test, respectively, in both WT and CB2-/- mice in the CPP test. Furthermore, the CB2 receptor reverse agonist SR144528 (3 mg/kg) inhibits morphine-evoked psychomotor stimulation at doses of 3 and 10 mg/kg, while increasing its rewarding effect in comparison to wild-type mice who received only a vehicle. These findings suggest that CB2 receptors are involved in the modulation of morphine-induced psychomotor stimulation and reward in mice. The inhibition of morphine-evoked psychomotor stimulation by SR144528 supports the role of CB2 receptors in regulating motor activity, possibly by modulating dopamine release in the mesolimbic pathway in particular dopamine release from VTA neurons (Pintori et al., 2021). These neurons are innervated by glutamatergic projections from the PFC, basolateral amygdala, and other structures that are all under the inhibitory control of the GABAergic interneurons of the VTA (Barrot et al., 2012). Morphine, through the MOR, inhibits the disinhibition of glutamatergic neurons on the VTA neurons, inducing an increased release of dopamine, activation of the basal ganglia circuit, and the development of an addictive memory by long-term potentiation of the neurons of the NAcc, PFC, amygdala, and hippocampus (Fields and Margolis, 2015). In a study conducted by Alavi et al., they found that O-1602 an atypical cannabinoid at the doses of 0.2, 1, and 5 mg/kg reduced the expression of morphine CPP with an increase in locomotor activity at the dose of 5 mg/kg (Alavi et al., 2016).

Furthermore, the observation that morphine on its own at a dose of 3 mg/kg did not induce a rewarding effect, but when administered to animals pre-treated with SR144528, it did induce reward, suggesting a complex interaction between CB2 receptors and the rewarding effects of morphine. This interaction may involve the modulation of dopamine release in the NAcc, a key brain region involved in reward processing (Li et al., 2021). It has been shown that CB2 receptors are colocalized with the dopamine D3 receptor at the level of the Nacc, CB2 receptors are also expressed at the level of glutamatergic projections of the VTA and it allows inhibition of these last ones, at the post-synaptic level it potentiates the M-type currents of K^+^. CB2 receptors are expressed in microglia, astrocytes, and dopaminergic neurons of the VTA that project into the Nacc (Sperlágh et al., 2009, Navarrete et al., 2013). Taking into account that the same structures involved in morphine addiction are involved in cocaine addiction, we can fully say that the inhibition of CB2 receptors causes the opposite of all that has been mentioned above. The reinforcing effect found in CB2-/- mice is therefore probably related to the loss of the modulatory effects of CB2 receptors on the reward circuit.

Interestingly a recent study By Reichenbach et al., showed that there was significant crosstalk between the CB2 receptor and morphine receptors, in this study it was found that CB2 agonist O-1966 had different proprieties in relationship to morphine efficiency following three treatment regimens (co-treatment, pretreatment with CB2 agonist and pretreatment with morphine), pretreatment with CB2 agonist and simultaneous treatment with morphine both negatively affected morphine potency and ended up speeding the tolerance development process, while pretreatment with morphine, had augmented morphine efficiency and delayed tolerance, moreover in the same study it was shown that MOR bidding affinity to DAMGO was reduced when O-1966 was first incubated and morphine after, but the authors are speculating whether this is because O-1966 is a negative allosteric modulator of the MOR, propriety that is shared by several cannabinoid ligands (Reichenbach et al., 2022).

An important number of people who take prescribed opioids do not develop problematic use for unknown reasons (Higgins et al., 2018). Deeper examinations of opioid abuse liability studies reveal large differences in individual abuse potential ratings (Bickel et al., 1989; Strain et al., 1992; Walsh et al., 1996). This means that in non/low vulnerable patients to addiction, morphine analgesia can be enhanced.

Taken together, this study suggests that the modulation of CB2 receptors exerts a significant influence on morphine activity, impacting both its analgesic effects and addictive potential. While the use of CB2 receptor antagonists alongside acute morphine treatments show promise in enhancing the analgesic properties of this opioid, chronic treatment with CB2 receptor negative modulation may strengthen the rewarding aspects of morphine, potentially exacerbating its addictive nature.

Further research is imperative to delve deeper into the intricate signaling mechanisms that underlie the interplay between cannabinoid and opioid receptors within the context of reward processing. Despite a wealth of data on the crosstalk between the endocannabinoid and opioid systems, there remain significant unresolved issues warranting further investigation While existing studies predominantly focus on the pharmacology of these receptors, there is a notable gap in research exploring the fundamental mechanisms governing the interaction between these physiological systems. Recent advancements, such as transcriptomic analyses in models like CB1 knockout mice with nerve pain, have revealed substantial alterations in the transcriptomic profile of dorsal root ganglion neurons, underscoring the need for continued exploration into the intricate relationships between cannabinoid and opioid receptors at a molecular level.

## CRediT authorship contribution statement

**Khalid Taghzouti:** Supervision, Resources, Project administration. **Oualid Abboussi:** Writing – review & editing, Validation, Supervision, Project administration, Methodology, Investigation, Funding acquisition, Formal analysis, Conceptualization. **Machich Omar:** Writing – original draft, Methodology, Investigation, Data curation, Conceptualization. **Safae Ouzzaouit:** Writing – review & editing, Writing – original draft, Investigation, Data curation, Conceptualization. **Meriem Moussafir:** Writing – review & editing, Methodology, Investigation, Data curation. **Souad Skalli:** Writing – review & editing.

## Conflicts of interest

We confirm that we have no financial or personal relationships with individuals or organizations that could bias our work. There are no conflicts of interest to report in relation to this manuscript.
